# MISEXPRESSION OF GENES LACKING CPG ISLANDS IS A SHARED TRAIT OF MAMMALIAN AGING

**DOI:** 10.1093/geroni/igac059.1069

**Published:** 2022-12-20

**Authors:** Samuel Beck

**Affiliations:** Boston University School of Medicine, Boston, Massachusetts, United States

## Abstract

Changes in the 3-D architecture of chromatin are observed in various diseases and are also a hallmark of aging. Disruption of the nuclear lamina and associated heterochromatin are commonly observed in various aging contexts, including premature aging diseases, cellular senescence, and normative aging. Although these conserved structural changes have been reported for over two decades, their impacts on transcription and contribution to age-related degenerative changes remain unknown. By performing a large-scale computational analysis and experimental validation, here we show that genes lacking CpG islands (CGI- genes), which form heterochromatin when transcriptionally silent, are globally misexpressed in aged nuclei with disrupted chromatin architectures. We demonstrate that CGI- gene misexpression is a common feature of mammalian aging and explains the molecular basis of various age-associated defects, ranging from loss of cellular identity and increased transcriptional noise to age-associated chronic inflammation. Our findings reveal that CGI- gene misexpression is directly associated with age-related physiological deterioration, thus providing a novel biomarker of aging.

